# Long-term effectiveness of Self-Help Plus in refugees and asylum seekers resettled in Western Europe: 12-month outcomes of a randomised controlled trial

**DOI:** 10.1017/S2045796022000269

**Published:** 2022-06-08

**Authors:** G. Turrini, M. Purgato, F. Tedeschi, C. Acartürk, M. Anttila, T. Au, K. Carswell, R. Churchill, P. Cuijpers, F. Friedrich, C. Gastaldon, T. Klein, M. Kösters, T. Lantta, M. Nosè, G. Ostuzzi, D. Papola, M. Popa, M. Sijbrandij, L. Tarsitani, L. Todini, E. Uygun, M. Välimäki, L. Walker, J. Wancata, R. G. White, E. Zanini, M. van Ommeren, C. Barbui

**Affiliations:** 1 WHO Collaborating Centre for Research and Training in Mental Health and Service Evaluation, Department of Neuroscience, Biomedicine and Movement Sciences, Section of Psychiatry, University of Verona, Verona, Italy; 2 Cochrane Global Mental Health, University of Verona, Verona, Italy; 3 Department of Psychology, Koc University, Istanbul, Turkey; 4 Faculty of Medicine, Department of Nursing Science, University of Turku, Turku, Finland; 5 Department of Mental Health & Substance Use, World Health Organization, Geneva, Switzerland; 6 Centre for Review and Dissemination, University of York, York, UK; 7 Department of Clinical, Neuro, and Developmental Psychology, Amsterdam Public Health Institute, and WHO Collaborating Centre for Research and Dissemination of Psychological Interventions, Vrije Universiteit Amsterdam, Amsterdam, The Netherlands; 8 Clinical Division of Social Psychiatry, Department of Psychiatry and Psychotherapy, Medical University of Vienna, Vienna, Austria; 9 Department of Psychiatry II, Ulm University, Günzburg, Germany; 10 Department of Primary Care and Mental Health, University of Liverpool, Liverpool, England; 11 Department of Human Neurosciences, Sapienza University of Rome, Rome, Italy; 12 Trauma and Disaster, Mental Health, Bilgi University, Istanbul, Turkey; 13 Xiangya Nursing School, Evidence-based Center, Central South University, Hunan, China; 14 Department of Health Sciences, University of York, York, UK; 15 School of Psychology, Queen's University Belfast, Belfast, Northern Ireland

**Keywords:** Prevention, psychosocial intervention, randomised controlled trial, refugees

## Abstract

**Aims:**

As refugees and asylum seekers are at high risk of developing mental disorders, we assessed the effectiveness of Self-Help Plus (SH + ), a psychological intervention developed by the World Health Organization, in reducing the risk of developing any mental disorders at 12-month follow-up in refugees and asylum seekers resettled in Western Europe.

**Methods:**

Refugees and asylum seekers with psychological distress (General Health Questionnaire-12 ⩾ 3) but without a mental disorder according to the Mini International Neuropsychiatric Interview (M.I.N.I.) were randomised to either SH + or enhanced treatment as usual (ETAU). The frequency of mental disorders at 12 months was measured with the M.I.N.I., while secondary outcomes included self-identified problems, psychological symptoms and other outcomes.

**Results:**

Of 459 participants randomly assigned to SH + or ETAU, 246 accepted to be interviewed at 12 months. No difference in the frequency of any mental disorders was found (relative risk [RR] = 0.841; 95% confidence interval [CI] 0.389–1.819; *p*-value = 0.659). In the per protocol (PP) population, that is in participants attending at least three group-based sessions, SH + almost halved the frequency of mental disorders at 12 months compared to ETAU, however so few participants and events contributed to this analysis that it yielded a non-significant result (RR = 0.528; 95% CI 0.180–1.544; *p*-value = 0.230). SH + was associated with improvements at 12 months in psychological distress (*p*-value = 0.004), depressive symptoms (*p*-value = 0.011) and wellbeing (*p*-value = 0.001).

**Conclusions:**

The present study failed to show any long-term preventative effect of SH + in refugees and asylum seekers resettled in Western European countries. Analysis of the PP population and of secondary outcomes provided signals of a potential effect of SH + in the long-term, which would suggest the value of exploring the effects of booster sessions and strategies to increase SH + adherence.

## Introduction

Refugees and asylum seekers are exposed to multiple stressors and traumatic events occurring in their country of origin, before migration, and during the migration process (Steel *et al*., 2009; Zimmerman *et al*., 2011; Giacco, 2019). During the post-migration phase, in the host country of resettlement, additional stressors and traumatic events may also frequently occur (Porter and Haslam, 2005; Jannesari *et al*., 2020; Wicki *et al*., 2021). Exposure to such stressors and repeated traumatic events may spread over long periods of time, and increases the risk of developing mental disorders (Barbui *et al*., 2020; Jannesari *et al*., 2020; Nosè *et al*., 2020; de Silva *et al*., 2021; Jowett *et al*., 2021).

In order to address the development of mental health problems and disorders, a number of studies assessed the effectiveness of a wide range of interventions designed to decrease psychological symptoms in refugees and asylum seekers (Nosè *et al*., 2017; Turrini *et al*., 2017, 2021; Thompson *et al*., 2018; Coventry *et al*., 2020; Jericho *et al*., 2021; Morina *et al*., 2021). Overall, meta-analyses of these studies showed a beneficial effect of psychosocial interventions, with three main caveats: first, effectiveness has been shown in the short-term only, while no long-term data are available; second, mainly treatment interventions were investigated whereas no studies were carried out to investigate the prevention of mental disorders; and third, the intervention delivery required extensive training and supervision, and intense implementation modalities, limiting their scale-up to only settings with high-resources.

In this context, the World Health Organization (WHO) developed Self-Help Plus (SH + ), a low-intensity self-help stress management programme aimed at reducing stress and improving overall wellbeing in people exposed to adversities (Epping-Jordan *et al*., 2016). SH + has been shown to be feasible and acceptable with refugees in Uganda (Tol *et al*., 2020), with positive findings indicating short-term improvements in psychological distress. Aiming to test whether SH + may have a preventative effect in asylum seekers and refugees, we designed two randomised trials, one in Western Europe and another in Turkey (Purgato *et al*., 2019). Both the Western European and Turkey studies showed evidence of an effect of SH + in preventing the onset of mental disorders and reducing stress, but differences were observed between the studies. The effect was much more pronounced for the Turkey study where efficacy (i.e. reducing the frequency of any mental disorder) was observed at 6 months (Acarturk *et al*., 2022), compared to the Western European study where a preventative effect was only found immediate post-intervention and not after 6 months (Purgato *et al*., 2021). In both studies, we planned a long-term follow-up at 12 months, but data at 12 months are only available for the Western European study due to logistic and administrative problems occurred in Turkey.

The aim of the present study is to report on the long-term effectiveness of SH + in reducing the risk of developing any mental disorder at 12-month follow-up in refugees and asylum seekers resettled in Western European settings. We additionally tested the long-term effects of SH + on a wide range of secondary outcomes, including psychological symptoms of depression and anxiety, functional impairment, wellbeing, perceived psychological problems, quality of life, and post-migration living difficulties (PMLD).

## Method

### Trial design

The trial was a rater-blind, parallel group, multinational RCT conducted in five countries (Austria, Finland, Germany, Italy, and two sites in the UK, namely England and Scotland), with outcome assessments at post-intervention and after 6 and 12 months of follow-up. We randomised participants to SH + or enhanced treatment as usual (ETAU) in a 1:1 ratio. We registered the trial protocol in clinicaltrials.gov (NCT03571347) before starting recruitment, and it was subsequently published in a scientific journal (Purgato *et al*., 2019). The study received ethical approval from the WHO Research Ethics Review Committee, and from the Ethics Committees of all participating sites. A detailed description of trial design, participant recruitment, intervention characteristics, and post-intervention and 6-month results, is available elsewhere (Purgato *et al*., 2021).

In each recruiting site, local organisations providing social, health, and/or legal support to refugees and asylum seekers were approached to identify potentially eligible participants. Based on a situational analysis of international migration flows, refugees and asylum seekers from Syria, Afghanistan, Pakistan, Iraq and Nigeria were identified as potential target groups (Purgato *et al*., 2021). All screening, baseline, and follow-up assessment questionnaires were administered in interview format [face-to-face in person or through secure videoconferencing tools, due to the severe acute respiratory syndrome coronavirus 2 (SARS-CoV2) pandemic] or self-administered after the participants signed informed consent forms. Assessors were trained in the administration of rating scales, instructed on how to perform follow-up assessments while preserving effective masking, and assisted by cultural mediators when needed.

### Participants

Adult refugees or asylum seekers with psychological distress, as assessed by the General Health Questionnaire 12-item (GHQ ⩾3) (Kilic *et al*., 1997), but who did not meet diagnostic criteria according to the Mini International Neuropsychiatric Interview (M.I.N.I.) (Sheehan *et al*., 1998), were eligible to be included in the study. Eligible participants were excluded if they: (a) had an acute medical condition contraindicating participation; (b) showed clinical evidence of imminent suicide risk or suicide risk scored as ‘moderate or high’ according to the M.I.N.I. (Sheehan *et al*., 1998); (c) presented signs of impaired decision-making as evidenced by responses during the clinical interview where ad-hoc questions were administered; (e) could not understand Dari, Urdu, Arabic, or English; (f) did not sign a written informed consent form. Refugees and asylum seekers who were excluded because of a diagnosis of a mental disorder and/or an imminent suicide risk were referred to professional treatment.

### Interventions

The intervention delivery lasted from September 2018 until March 2020. The SH + programme was developed by WHO, as described elsewhere, and is now publicly available (Epping-Jordan *et al*., 2016; World Health Organization, 2021). SH + consists of a pre-recorded audio course, delivered by briefly trained facilitators in a group setting and complemented with an illustrated self-help book adapted for the cultural groups included in the study. The book has been recently updated and published by WHO as *Doing What Matters in Times of Stress* (World Health Organization, 2020).

The pre-recorded audio format of SH + is innovative in that it seeks to ensure that key intervention components are delivered as intended without the burden of extensive facilitator training. The SH + programme is based on acceptance and commitment therapy (ACT), a form of cognitive-behavioural therapy (Hayes *et al*., 1999). ACT emphasises learning new ways to accommodate difficult thoughts and feelings while guiding people to take proactive steps toward living in a way that is consistent with their values (Epping-Jordan *et al*., 2016). Rather than trying to control the content of thinking and emotions, ACT aims to help individuals change their relationship to these events: the goals are not an explicit replacement of previous unhealthy psychological events with new and healthy events, but the cultivation of acceptance toward of the occurrence of unhealthy psychological events, defusion from strict adherence to those events and the committed action of behaviours that support living in ways that serve predetermined healthy values.

The SH + pre-recorded audio material was delivered across five 2-h sessions to groups of up to 30 people. The audio material imparts key information about stress management and guides participants through individual exercises and small group discussions. To augment the audio recordings, an illustrated self-help book reviews all essential content and concepts.

SH + was culturally adapted to the different target population groups following a protocol developed by WHO with the aim of developing adaptations in such a way that the language was suitable for as many dialects or ethnicities as possible and arriving at materials that was broadly acceptable, understandable to ensure that SH + could be sustainably delivered and potentially scalable. It was fully delivered in the native language of participants by trained facilitators with a migration background, who were native speakers of the target languages. Most facilitators had no prior work experience in this field and/or formal mental health training. Facilitators completed four to five days of training, which included listening to the audio recordings, receiving instruction in SH + facilitation skills, and role-playing and practising SH + sessions. Facilitators conducted additional practice groups before running sessions with participants as needed. The facilitator's role consisted of playing the audio, responding to questions, ensuring safe and smooth running of the group, demonstrating the exercises, and reading out scripted discussion questions.

Intervention supervision was provided by clinical psychologists or other health care professionals, who were available for questions, discussion and debriefing after the sessions. If necessary, additional training and consultation were available from SH + expert trainers at WHO through local visits. Fidelity was checked by the intervention supervisor through session adherence forms completed by facilitators. In addition, the intervention supervisor (a clinical psychologist, or a psychiatrist, or a specialised nurse with formal background in mental health) observed at least 10% of the sessions and completed an adherence form for each SH + session.

ETAU was provided to the control group, and consisted of routinely delivered social support and/or health care according to local regulations (Appendix, Table 6). Additionally, participants in the ETAU arm received baseline and follow-up assessments according to the study schedule (around 2 months and 6 months after randomisation), information about freely available health and social services, and links to community networks providing support to refugees and asylum seekers.

An Ethics Advisory Board (EAB), consisting of experts giving advice on any ethical issues related to the trial, supervised the ethical aspects of the study.

### Randomisation and masking

Randomisation was centralised and coordinated by the WHO Collaborating Centre of the University of Verona. The randomisation schedule was generated by the electronic software Castor Electronic Data Capture (EDC) (Castor EDC, 2017). Research team members involved in recruitment and randomisation were not able to access the randomisation list, and were not aware of the block size. Participants and facilitators could not be masked to treatment allocation, but outcome assessors and the statistician performing the analyses were kept blind to the allocation. The trial statistician was not involved in determining participants' eligibility, administering the intervention, measuring the outcomes and entering data.

### Outcomes

The primary outcome of the trial was the presence of current mental disorders at 6-month follow-up (Purgato *et al*., 2021). In this study report, we sought to investigate the frequency of mental disorders after 12 months of follow-up using the M.I.N.I. (Sheehan *et al*., 1998). Validation and reliability studies have been conducted comparing the M.I.N.I. to the Structured Clinical Interview for DSM-III-R (Spitzer *et al*., 1990) and the Composite International Diagnostic Interview, a structured interview developed by the World Health Organization. The results of these studies showed that the M.I.N.I. had similar reliability and validity properties, but can be administered in a much shorter period of time than the above-referenced instruments (Janca *et al*., 1995).

As secondary outcomes, the following measures were analysed at 12 months after randomisation: psychological distress assessed by the GHQ-12 questionnaire (Kilic *et al*., 1997); post-traumatic stress disorder (PTSD) symptoms assessed with the PTSD Checklist for DSM-5 (PCL-5) (Blanchard *et al*., 1996; Weathers *et al*., 2013); depressive symptoms assessed with the Patient Health Questionnaire-9 (PHQ-9) (Kroenke *et al*., 2001); self-defined psychosocial goals through questions exploring participants' problems and how they affect them, using the Psychological Outcome Profiles instrument (PSYCHLOPS) (Ashworth *et al*., 2012); functional impairment and subjective wellbeing as measured by the WHO Disability Assessment Schedule 2.0 (WHODAS) (World Health Organization, 2010) and the WHO-5 Wellbeing Index (WHO-5) (Heun *et al*., 1999), respectively; the general health status assessed using the EQ-5D-3L questionnaire (EuroQol Group, 1990); daily and environmental stressors were collected with the 17-item Checklist for PMLD (Riley *et al*., 2017). In addition, we assessed the frequency of health service use through the adapted version of the Client Socio-Demographic and Service Receipt Inventory, European Version (CSSRI-EU) (Chisholm *et al*., 2000). Adverse events reported spontaneously by the participants or observed by the research staff were recorded, reviewed by the EAB in regular meetings and reported to the WHO Ethics Committee in mid-term reports on a regular basis.

Researchers involved in screening, baseline, and follow-up assessments received specific two-hour training sessions for administering the M.I.N.I. (Sheehan *et al*., 1998) and the rating scales for measuring secondary outcomes by expert trainers based at the University of Verona.

### Statistical analysis

The 12-month analysis was pre-planned and reported in the research study protocol, but a sample size calculation was not performed as the primary outcome was set at 6 months. Descriptive statistics (mean and s.d. for continuous variables and absolute numbers and percentages for dichotomous variables) were computed on sociodemographic, premigration, migration and postmigration variables at baseline, and for clinical variables. A standardised mean difference (SMD) with values above 0.1 or below −0.1 was considered as an imbalance between treatment groups.

Both intention-to-treat analysis (including all participants with available data at baseline) and per protocol (PP) analyses (including participants who attended at least three SH + sessions) were carried out both for the M.I.N.I. and for continuous outcomes.

We calculated the proportion of participants with a current diagnosis of any mental disorders at 12 months, as assessed with the M.I.N.I. (Sheehan *et al*., 1998). We also estimated the proportion of participants who were M.I.N.I. positive in at least one timepoint (post-intervention, 6 months, 12 months), as a proxy of 12-month incidence of any mental disorder, as well as the proportion of participants who were M.I.N.I. positive at all timepoints, as a proxy measure of the persistence of mental disorders. In the case of individuals with missing data in one or two timepoints, in order to properly take the information related to their available M.I.N.I. values into account, we performed multiple imputations with chained equations through logistic regression, using the outcome values at the other timepoints as predictors, separately for each treatment arm and for each analysis. The number of imputed samples was determined by following the quadratic rule described by von Hippel (von Hippel, 2020). In particular, 10 samples were tried first and then analyses were repeated using the lowest number of needed samples to fulfil the rule estimated by the Stata ‘how_many_imputations’ command (von Hippel, 2018), rounded to the nearest multiple of 10 above.

Binary outcomes were compared between the two groups through the risk ratio (RR) and its 95% confidence interval (CI). For secondary continuous outcomes, a mixed analysis of covariance (ANCOVA) controlling for baseline scores was performed, with robust standard errors and distinct variances for the three timepoints after baseline. In addition to mixed models, a last observation carried forward (LOCF) approach was implemented. Standardised coefficients, together with their standard errors, were also calculated with the Stata ‘stdBeta’ command (Hemken, 2016). We tested the null hypothesis of the intervention having no effect on any outcomes versus the alternative hypothesis of the intervention having an effect on at least one outcome by performing a seemingly unrelated regression (SUR) (Zellner, 1962) equations model (in its modification to allow for unbalanced data proposed in Baum and Schaffer (Baum and Schaffer, 2009) through the Stata ‘suregub’ command). In particular, SURs were performed for each time point, controlling for baseline values. For each questionnaire, in case of missing items, we used the Corrected Item Mean Substitution method [i.e. the item mean across participants weighted by the subject's mean of completed items (Huisman, 1999)], using information from subjects belonging to the same treatment arm for the same follow-up time, through the Stata ‘hotvalue’ command (Millar, 2008). The substitution was only performed if resulting in admissible values in all cases, and only for observations having less than 50% of the missing item. As a sensitivity analysis, we reran our models for the scores derived as the sum of single items of the scale without any data imputation. Participants lost to follow-up were also compared, with respect to the clinical and the main sociodemographic and migration characteristics, to those who reached the 12-month follow-up.

For all outcomes, possible interactions between intervention and specific variables (recruiting centre, country of origin, gender, age, years of education and length of stay in the hosting country) were evaluated. In particular, in the case of continuous outcomes, SUR for unbalanced data on all outcomes using the LOCF dataset was performed, with their value at baseline, treatment status, all potential moderators, and their interactions with treatment status, as predictors. A global test on all interaction terms was implemented and, in case of statistical significance, the same test was performed for each scale. Finally, for scales meeting the statistical significance threshold, single regressions were considered.

In the case of binary outcomes, in order to avoid the issue of poor performance of the model in case of solutions near the boundary described in Zhu and colleagues (Zhu *et al*., 2018) possible moderators were considered separately. In particular, Poisson regression models with robust standard errors and, in case of quasi-separation (i.e., empty cells), Firth logistic regressions (Firth, 1993) were performed, in both cases with the variable ‘intervention allocation’, each variable separately, and its interaction with treatment as regressors, using the Bonferroni correction to take multiple testing into account.

Both for the M.I.N.I. and for continuous outcomes, we performed multivariate analyses to take confounding factors into account, again including the baseline value as a covariate; the LOCF dataset was considered for continuous outcomes, while the Poisson regression model, with a robust error variance was adopted for binary outcomes.

In all regressions using the LOCF approach, White's test for homoskedasticity against unrestricted forms of heteroskedasticity was used to assess whether including robust standard errors or not (White, 1980).

Frequency of health service use was also compared between the two groups. Statistical significance of the differences was evaluated through Chi-square test or Fisher's exact test, as appropriate. The statistical analysis was masked, and the statistician was blinded to the intervention groups until the completion of the analysis. All analyses were performed using Stata 17 (StataCorp, 2021).

## Results

**Fig. 1.**
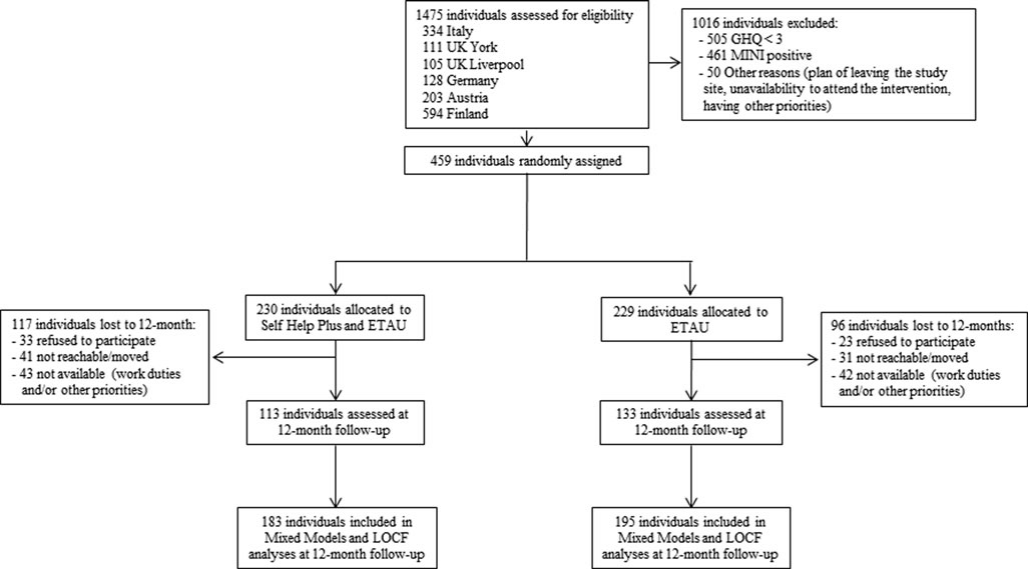
CONSORT flow diagram.

After screening of 1475 potentially eligible participants, 459 met the inclusion criteria, consented to be randomised by signing a written informed consent form, and were allocated to either SH + (230) or ETAU (229) (Fig. 1).

After 12 months of follow-up we could assess 246 participants (54%). Participants were lost to 12-month follow-up because they moved to other locations (*n* = 72), refused to participate (*n* = 56), or were not available due to other personal priorities (i.e., working, housing, other) (*n* = 85). The 213 participants lost to follow-up were similar, in terms of sociodemographic and clinical characteristics, to those who reached the 12-month follow-up, with the exception of country of origin, number of relatives and children, and severity of psychological distress (Appendix, Table 5). Specifically, participants lost to follow-up showed a higher level of psychological distress (SMD = 0.133); participants from Afghanistan were less represented (SMD = −0.118), while there were more participants from Pakistan (SMD = 0.129), with fewer relatives they lived with (SMD = −0.137) and children (SMD = −0.118). The drop-out rate did not significantly differ between SH + and ETAU (RR = 1.213; CI 0.995–1.480; *p*-value = 0.056). The two groups were well balanced for most sociodemographic characteristics both at baseline (459 participants) and at 12-month follow-up (246 participants). The full list of sociodemographic characteristics for participants who reached the 12-month follow-up is presented in the Appendix (Table 1), while selected sociodemographic characteristics of the randomised participants are shown in Table 1.

**Table 1.** Socio-demographic characteristics

Variable	ETAU	SH +	Difference (SE)	SMD
Mean age (s.d.), years	31.537 (9.505)	32.961 (10.779)	−1.424 (0.949)	−0.099
Female gender (%)	30.13 (69/229)	28.26 (65/230)	−0.019 (0.043)	−0.029
Mean education (s.d.), years	10.157 (5.451)	10.452 (4.890)	−0.295 (0.497)	−0.040
Type of education (%)				
Illiterate	11.50 (26/226)	5.73 (13/227)	**−0.058 (0.026)**	**−0.146**
Primary school	40.71 (92/226)	44.93 (102/227)	−0.042 (0.047)	−0.060
High school	27.88 (63/226)	27.75 (63/227)	−0.001 (0.042)	−0.002
University	19.91 (45/226)	20.70 (47/227)	−0.008 (0.038)	−0.014
Mean relatives (s.d.), *n*	1.655 (2.413)	1.409 (1.978)	−0.246 (0.206)	−0.079
Mean children (s.d.), *n*	1.369 (2.426)	1.348 (1.798)	−0.021 (0.200)	−0.007
Country of origin (%)				
Afghanistan	14.41 (33/229)	14.35 (33/230)	−0.001 (0.033)	−0.001
Iraq	18.78 (43/229)	17.39 (40/230)	−0.014 (0.036)	−0.025
Nigeria	24.02 (55/229)	25.65 (59/230)	−0.016 (0.040)	−0.027
Pakistan	09.61 (22/229)	8.26 (19/230)	−0.013 (0.027)	−0.033
Syria	28.38 (65/229)	28.26 (65/230)	−0.001 (0.042)	−0.002
Other country	4.80 (11/229)	06.09 (14/230)	−0.013 (0.021)	−0.040
Travel route (%)				
Balkan	34.50 (79/229)	28.26 (65/230)	−0.062 (0.043)	−0.095
Eastern	19.65 (45/229)	22.17 (51/230)	−0.025 (0.038)	−0.044
African	26.64 (61/229)	27.83 (64/230)	−0.012 (0.042)	−0.019
Other	18.34 (42/229)	20.43 (47/230)	−0.021 (0.037)	−0.037
Detention during transition (%)	29.46 (66/224)	31.70 (71/224)	−0.022 (0.044)	−0.034

Values in bold indicate an imbalance at baseline.

The full list of sociodemographic characteristics for participants included at baseline are presented elsewhere.(Purgato *et al*., 2021) Briefly, the mean age was 32.96 years (s.d. = 10.779) in the SH + group and 31.53 years (s.d. = 9.505) in the ETAU group, with one-third of women in both groups. For both groups, the average length of education was about 10 years, with primary school as the most frequent level of education. Almost one-third of the participants came from Syria, and one fourth from Nigeria, followed by Iraq, Afghanistan, Pakistan and other countries. Almost 30% were detained during the migration process.

Assessment of more than 10% of SH + sessions showed that all the components of the intervention were delivered in line with the manual. Observation of the sessions by the supervisors showed an almost perfect fidelity, with the exception of more time taken to restart the audio and for group discussions than allotted in the manual. Three adverse events (2 in ETAU and 1 in SH + ) were registered at 12-months follow up and judged as not related to the study participation by the EAB. In addition to SH + sessions or ETAU, participants received minimal health care during the study period, which did not differ between the two groups (Appendix, Table 6).

Differences between study conditions on primary and secondary outcomes are reported in Table 2.

**Table 2.** Summary statistics of results for primary and secondary outcomes at baseline and 12-month follow-up

Binary outcomes	ETAU	SH +	RR (CI)	*p*-value
Frequency of current mental disorders				
12 months – Intention-to-treat	14/133 (10.53%)	10/113 (8.85%)	0.841 (0.389; 1.819)	0.659
12 months – Per Protocol analysis	14/133 (10.53%)	4/72 (5.56%)	0.528 (0.180; 1.544)	0.230
Incidence of any mental disorders				
12 months – Intention-to-treat	58/229^a^ (25.46%)	57/230^a^ (24.68%)	0.969 (0.676; 1.391)	0.865
12 months – Per Protocol analysis	58/229^a^ (25.46%)	23/117^a^ (19.94%)	0.783 (0.482; 1.273)	0.324
Persistence of any mental disorders				
12 months – Intention-to-treat	14/229^a^ (6.10%)	2/230^a^ (1.08%)	0.178 (0.026; 1.220)	0.079
12 months – Per Protocol analysis	14/229^a^ (6.10%)	1/117^a^ (1.24%)	0.204 (0.027; 1.513)	0.120
Continuous outcomes	Mean (s.d.)	Mean (s.d.)	Coefficient	*p*-value
GHQ score (0–12)				
Screening (*n* = 459)	5.507 (2.447)	5.619 (2.185)	–	–
12 months (*N* = 374): Mixed model	2.930 (3.298)	1.782 (2.488)	**−1.026 (−1.726; −0.326)**	**0**.**004**
12 months LOCF (*N* = 374)	3.073 (3.312)	2.456 (3.020)	−0.567 (−1.183; 0.050)	0.072
PCL5 score (0–80)				
Baseline (*n* = 459)	22.765 (16.239)	24.692 (16.352)	–	–
12 months (*N* = 373): Mixed model	14.606 (15.306)	12.450 (15.298)	−3.219 (−6.579; 0.142)	0.061
12 months LOCF (*N* = 373)	16.350 (15.023)	15.040 (15.615)	−2.016 (−4.839; 0.807)	0.161
PHQ9 score (0–27)				
Baseline (*n* = 459)	8.384 (5.546)	8.478 (5.816)	–	–
12 months (*N* = 372): Mixed model	5.810 (6.045)	4.028 (4.522)	**−1.608** (**−2.850; −0.367)**	**0**.**011**
12 months LOCF (*N* = 372)	6.335 (5.917)	5.055 (5.484)	**−1.193** (**−2.295; −0.092)**	**0**.**034**
WHO-5 score (0–100)				
Baseline (*n* = 459)	47.354 (24.984)	46.723 (23.625)	–	–
12 months (*N* = 373): Mixed model	52.157 (27.248)	62.111 (23.435)	**10.051** (**4.073; 16.028)**	**0**.**001**
12 months LOCF (*N* = 373)	50.333 (26.673)	58.961 (24.050)	**8.940** (**3.903; 13.976)**	**0**.**001**
WHODAS score (0–1)				
Baseline (*n* = 459)	0.152 (0.144)	0.148 (0.141)	–	–
12 months (*N* = 373): Mixed model	0.088 (0.107)	0.072 (0.107)	−0.017 (−0.040; 0.006)	0.144
12 months LOCF (*N* = 373)	0.092 (0.123)	0.077 (0.104)	−0.013 (−0.034; 0.007)	0.197
PSYCHLOPS score (0–20)				
Baseline (*n* = 432)	13.925 (4.396)	14.097 (4.013)	–	–
12 months (N = 350): Mixed model	10.758 (5.431)	10.060 (5.603)	−0.647 (−2.016; 0.722)	0.354
12 months LOCF (N = 350)	10.959 (5.432)	10.547 (5.595)	−0.539 (−1.632; 0.553)	0.332
PMLD score (0–68)				
Post-intervention^b^ (*n* = 342)	25.587 (12.846)	24.174 (12.199)	–	–
12 months (*N* = 288): Mixed model	17.811 (11.833)	17.428 (12.253)	0.123 (−2.518; 2.765)	0.927
12 months LOCF (*N* = 288)	18.844 (11.489)	18.886 (12.061)	0.164 (−2.085; 2.413)	0.886
EQ-5D-3L				
Baseline (*n* = 455)	0.706 (0.276)	0.716 (0.280)	–	–
12 months (*N* = 315): Mixed model	1.460 (1.542)	1.336 (1.607)	−0.125 (−0.544; 0.294)	0.558
12 months LOCF (*N* = 315)	1.277 (1.371)	1.170 (1.400)	−0.099 (−0.384; 0.186)	0.493

Values in bold highlight statistically significant differences. *n* indicates the number of observations at the first available measurement, while *N* represents the number of individuals used in regression.

a Numbers are not observed but estimated.

b Baseline not measured.

**Fig. 2.**
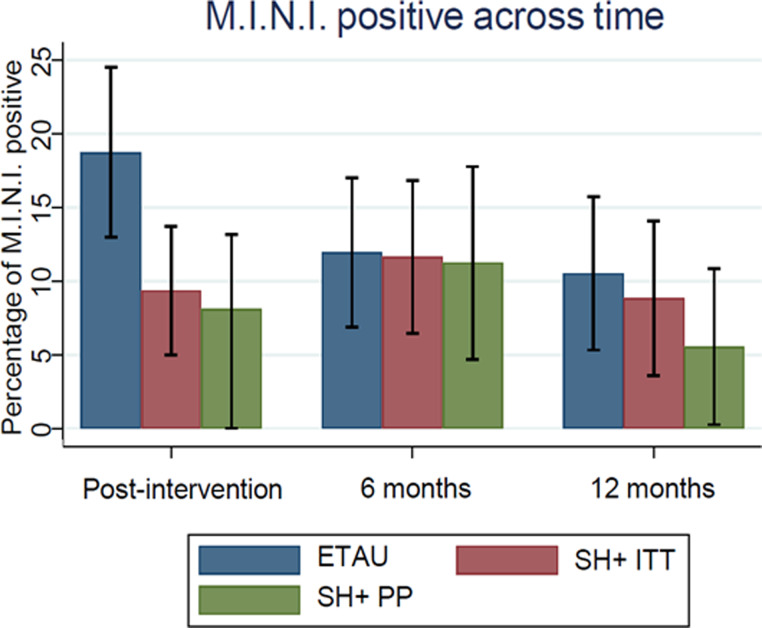
Trend in the frequency of any mental disorders over time. SH + , Self-Help Plus; ETAU, Enhanced Treatment as Usual; PP, Per Protocol; ITT, Intention to Treat.

The ITT analysis of the primary outcome revealed that SH + was not associated with a significant reduction in the frequency of any current mental disorders as measured with the M.I.N.I. at 12 months (RR = 0.841; 95% CI 0.389–1.819; *p*-value = 0.659). Similarly, no significant differences between SH + and ETAU were detected in terms of incidence (*p*-value 0.865) or persistence (*p*-value 0.079) of any mental disorders (Table 2). In the PP analyses, SH + almost halved the frequency of mental disorders at 12 months compared to ETAU, however so few participants and events contributed to this analysis that it yielded a non-significant result (RR = 0.528; 95% CI 0.180–1.544; *p*-value = 0.230) (Table 2, and Fig. 2). The majority of detected mental disorders at 12 months were major depressive disorders (8/113 participants in the SH + group and 9/133 participants in the ETAU). We also detected anxiety disorders (1/113 participants in the SH + group and 4/133 participants in the ETAU) and PTSD disorders (1/113 participants in the SH + group and 2/133 participants in the ETAU), together with one occurrence of antisocial personality disorder in the SH + group, and one of alcohol use disorder in the ETAU.

With respect to continuous outcomes, the mixed-models analyses revealed that SH + compared to ETAU was associated with improvements at 12-months for psychological distress (GHQ-12, *p*-value = 0.004), depressive symptoms (PHQ-9, *p*-value = 0.011), and wellbeing (WHO-5, *p*-value = 0.001) (Table 2), while the LOCF analysis showed a beneficial effect of receiving SH + on depressive symptoms and wellbeing, but not on psychological distress. The PP analysis of continuous outcomes confirmed the beneficial effect of SH + on psychological distress, depressive symptoms and wellbeing, both for the mixed-model and for the LOCF approach (Appendix, Table 2).

Secondary analyses of continuous outcomes conducted without any imputation of missing values, did not identify any relevant difference with respect to the main mixed-model analyses (Appendix, Table 3). Results of the LOCF analyses controlling for variables showing imbalance at baseline confirmed a significant effect of SH + versus ETAU in lowering PHQ-9 scores and increasing WHO-5 ones (Appendix, Table 4).

Heterogeneity of the effect of treatment on outcomes was investigated by testing for interactions between intervention allocation and potential moderators. None of the interactions reached a statistical significance for binary outcomes.

By performing SURs on secondary outcomes, a global test on all interactions of the variable ‘intervention allocation’ with the potential moderators on all regressions, turned out to be significant (*p*-value = 0.004). Specifically, single regressions revealed the global statistical significance of all interactions only in the regression having PSYCHLOPS as an outcome (*p*-value = 0.042). This analysis found that only study centre (*p*-value = 0.029) and gender (*p*-value = 0.008) emerged as statistically significant moderators (Appendix, Figure 1 and Fig. 2). In particular, a significant protective effect of SH + on the PSYCLOPS score at 12-months emerged for people recruited in York and Ulm study sites and for women.

## Discussion

To our knowledge, this is the first study in refugees and asylum seekers that examined the long-term effects of a preventive intervention on the development of mental disorders. Even though it did not show any effect of SH + in preventing the onset of mental disorders at 12-month follow-up, in the PP population, that is in participants who attended at least three out of five SH + sessions, the proportion of refugees and asylum seekers with a mental disorder was halved by the experimental intervention. Additionally, the estimated persistence was lower for SH + as compared with ETAU. However, the absolute number of cases in these analyses was low, and no statistically significant difference was observed, indicating that no conclusions can be drawn by these figures. Nevertheless, they suggest that SH + may have positive long-term prevention effects, provided a sufficient dose of SH + is received, although this will require exploration in further research. In agreement with this interpretation, we identified a beneficial effect of SH + on psychological distress, depression symptoms and wellbeing at 12-month follow-up. In particular, the results on well-being were consistent over time (Purgato *et al*., 2021), which seems in line with the rationale of the intervention itself, which has a primary focus on tackling adversities by improving well-being. Also, it is important to point out that subsyndromal psychological distress is highly prevalent in refugee populations. Psychological distress poses risk for subsequent mental disorders and causes marked impairment, so a beneficial effect of SH + on psychological distress represents an important finding. A trial with a design similar to the present study conducted with refugees in Turkey found that participants allocated to SH + were significantly less likely to have any mental disorders after 6 months of follow-up compared to the control group (Acarturk *et al*., 2022). Considering that both this study and the Turkish SH + study observed effects on key measures several months after the intervention, we hypothesise these findings may be related to the main SH + components which were delivered over the five weekly sessions. However, this hypothesis requires further research considering also the results presented by Purgato and colleagues (Purgato *et al*., 2021) that found a significant difference in favour of SH + in reducing the frequency of current mental disorders in refugees and asylum seekers resettled in Western Europe, only immediately after the intervention.

We acknowledge some study limitations. A major challenge was that a considerable proportion of randomised participants declined to be involved in the study anymore at different stages, and refused to complete the follow-up assessments. Interestingly, this may be related to the type of population included, who were participants without a mental disorder, given the preventative aim, but at risk of developing one, with several social and everyday life priorities and potentially poorer recognition or less time to make their mental health a priority (Byrow *et al*., 2020). Indeed, most participants declining follow-up interviews reported that competing stressors and other personal priorities including housing, unstable working conditions, management of visa issues, the safety of family members, a fear of being returned to home country, plans to move to another country or even having moved to another location, did not allow them to be engaged in follow-up assessments. In terms of study design, losses to follow-up may be problematic for two main reasons. First, while the experimental and control group were comparable at baseline in terms of socio-demographic and clinical characteristics, comparability at the endpoint of the study could be an issue. For example, it could be that the cases lost to follow-up did not show up for the assessments due to clinical reasons that might bias the trial results, as a significant increase of psychological distress. At this regard, we checked the comparability of the two groups at 12 months, which demonstrated that participants completing the 12-month follow-up assessments were mostly similar in terms of socio-demographic characteristics. Moreover, ‘lost to follow-up’ participants were similar to completers, thus suggesting that these losses did not affect results relevantly. Second, the study lacked sufficient statistical power, as the lack of a relevant proportion of participants decreased the possibility of detecting significant differences, especially for the primary outcome.

A second challenge was the lack of a double-blind design, which was not feasible due to the nature of the intervention, with a risk of performance and detection bias. Likely, performance bias did not occur, as the two groups received the same type and amount of social and health care interventions, and detection bias was limited by the employment of masked outcome assessors. A third challenge was that the SARS-CoV2 pandemic impacted the study procedures, because follow-up assessments were conducted using online tools instead of in-person meetings in both SH + and ETAU in all recruiting sites. Although this switch influenced both arms equally, it is unknown whether this may have impacted the responses of participants, that may have felt less engaged or motivated. A fourth limitation refers to the fact that, even though several studies documented that a careful and culturally appropriate use of available instruments is feasible and allows a systematic recognition of psychological distress and psychiatric diagnoses (Acarturk *et al*., 2021) formal studies on use of these tools in refugee groups are lacking.

Despite these limitations, the present study has several strengths. First, the focus on a population that is epidemiologically and socially relevant (Turrini *et al*., 2017) but has seldom been included in randomised trials with long-term outcome evaluations. Second, the exclusion of participants with a mental disorder at baseline, making this study the only example of a trial with a truly preventative design in this vulnerable group. Third, the assessment of a WHO developed, low-intensity intervention, that is open access and has the potential for use at scale, as it can be delivered to large groups by non-specialist providers with minimal training, makes it relevant as a potential public health strategy to prevent mental disorders. Finally, the choice of a primary outcome that is fully consistent with a preventative design, namely the frequency of mental disorders as assessed with validated measures, along with a long-term outcome assessment that has almost never been attempted in such difficult-to-engage vulnerable populations, suggests its relevance to the evidence base on prevention.

The signals of a potential long-term effect observed in the present study may provide grounds for the design of new studies with long-term follow-up assessments. Strategies to minimise the risk of attrition bias should be developed, considering that asylum seekers and refugees are a difficult-to-follow population, often moved from one reception site to another, and often burdened by a number of challenging concerns that are given priority over mental health. As compared with the population included in the present study, future preventative studies should consider the inclusion of population groups at higher risk of developing mental disorders, as this would have advantages both in terms of chances to detect differences, as the frequency of the outcome of interest would increase, and in terms of practical implications, as SH + would be more easily implemented to selected groups of high-risk asylum seekers and refugees. These studies should additionally consider, in view of the experience gained in the present study, whether booster sessions, delivered after the group intervention, administered face-to-face or online, might assist in maintaining, corroborating, or even boosting its benefits over time. As a second aspect, strategies to optimise adherence to SH + sessions should be developed, and reasons for not attending the sessions better explored, as we hypothesise that its efficacy is closely related to attending the sessions as expected by the SH + manual (i.e. at least 3 out of 5 sessions). Moreover, new studies might explore its use in a stepped-care or collaborative care format with more intensive treatment for people in need at a later stage. Finally, new care models could deepen the importance of group-based intervention which seems to be promising, as they could address both social isolation and advocating for the rights and material needs of refugees by allowing the communities to interact. This may also help to address post-migration conditions usually associated with poorer mental health outcomes.

Overall, the present study failed to show any long-term preventative effect of SH + in refugees and asylum seekers resettled in Western European countries. SH + may provide an important contribution to the psychological wellbeing of refugees and asylum seekers, but its longer-term effects on the prevention of mental disorders requires further research, and more is required to address the social determinants of mental health in this population.

## Data Availability

Data supporting our findings are available in Appendix.

## Supplementary material

For supplementary material accompanying this paper visit https://doi.org/10.1017/S2045796022000269. click here to view supplementary material
