# Supplementary material for: Long-term effectiveness of Self-Help Plus in refugees and asylum seekers resettled in Western Europe: 12-month outcomes of a randomised controlled trial
Source: Epidemiol Psychiatr Sci. 2022 Jun 8;31:e39. doi: 10.1017/S2045796022000269 (PMC9220789; doi:10.1017/S2045796022000269)
Supplement: Supplementary file 1 [file S2045796022000269sup001.docx]

**Supplementary appendix**

**Long-term effectiveness of Self-Help Plus in refugees and asylum seekers resettled in Western Europe: 12-month outcomes of a randomized controlled trial**

**TABLE OF CONTENTS**

[**Table 1: Balance of 12 months full list of collected socio-demographic and clinical variables at baseline** 3](#_Toc95487954)

[**Table 2. Per-protocol analysis** 6](#_Toc95487955)

[**Table 3. Analysis with no imputation** 7](#_Toc95487956)

[**Table 4. Results controlling for imbalances at baseline** 7](#_Toc95487957)

[**Table 5. Balance of clinical and sociodemographic variables between Lost-to-follow-up (LTFU) and non-LTFU (completers)** 7](#_Toc95487958)

[**Table 6. Frequency of health service use during the study** 9](#_Toc95487959)

[**Test of interactions** 9](#_Toc95487960)

[**List of adverse events** 11](#_Toc95487961)

[**CONSORT 2010 checklist of information to include when reporting a randomised trial** 12](#_Toc95487962)

# **Table 1: Balance of 12 months full list of collected socio-demographic and clinical variables at baseline**

| **Variable** | **ETAU** | **SH+** | **Difference (SE)** | **Standardized Mean Difference** |
| --- | --- | --- | --- | --- |
| Age in years:  Mean (SD) | 31.782(9.123) | 33.681(10.588) | 1.899(1.257) | **0.136** |
| Female gender | 37/133 | 31/113 | -0.004 | -0.006 |
|  | (27.82%) | (27.43%) | (0.057) |  |
| ***Country of origin*** |  |  |  |  |
| Afghanistan | 25/108 | 17/113 | -0.038 | -0.071 |
|  | (18.80%) | (15.04%) | (0.048) |  |
| Iraq | 22/133 | 24/113 | 0.047 | 0.085 |
|  | (16.54%) | (21.24%) | (0.050) |  |
| Nigeria | 30/133 | 29/113 | 0.031 | 0.051 |
|  | (22.56%) | (25.66%) | (0.055) |  |
| Other country | 6/133 | 5/113 | -0.001 | -0.003 |
|  | (4.51%) | (4.42%) | (0.027) |  |
| Pakistan | 11/133 | 5/113 | -0.038 | **-0.111** |
|  | (8.27%) | (4.42%) | (0.032) |  |
| Syria | 39/133 | 33/113 | -0.001 | -0.002 |
|  | (29.32%) | (29.20%) | (0.058) |  |
| Single | 50/132 | 46/113 | 0.028 | 0.041 |
|  | (37.88%) | (40.71%) | (0.063) |  |
| Married/Cohabitant | 69/132 | 60/113 | 0.008 | 0.012 |
|  | (52.27%) | (53.10%) | (0.064) |  |
| Divorced widowed | 12/132 | 7/113 | -0.029 | -0.077 |
|  | (9.09%) | (6.19%) | (0.034) |  |
| N of relatives:  Mean (SD) | 1.902 (2.602) | 1.522 (1.955) | -0.380(0.298) | **-0.117** |
| N of children:  Mean (SD) | 1.569 (2.776) | 1.469 (1.691) | -0.100(0.300) | -0.031 |
| Years of education: Mean (SD) | 9.762 (5.400) | 10.703 (5.405) | 0.941 (0.712) | **0.123** |
| Illiterate | 16/131 | 7/111 | -0.059 | **-0.144** |
|  | 12.21% | 6.31% | (0.038) |  |
| Primary | 53/131 | 66/111 | 0.001 | 0.001 |
|  | 40.46% | 40.54% | (0.064) |  |
| High School | 38/131 | 26/111 | -0.056 | -0.090 |
|  | 29.01% | 23.42% | (0.057) |  |
| University | 24/131 | 31/111 | 0.096 | **0.161** |
|  | 18.32% | 27.93% | (0.054) |  |
| N of siblings | 4.342 (3.224) | 5.037 (3.084) | 0.696(0.420) | **0.156** |
| ***Religion*** |  |  |  |  |
| Christian | 38/132 | 28/111 | -0.036 | -0.057 |
|  | 28.79% | 25.23% | (0.057) |  |
| Muslim | 86/132 | 75/111 | 0.024 | 0.036 |
|  | 65.15% | 67.57% | (0.061) |  |
| Other religion | 8/132 | 8/111 | 0.011 | 0.032 |
|  | 6.06% | 7.21% | (0.032) |  |
| Single pre-migration | 50/132 | 46/113 | 0.028 | 0.041 |
|  | 37.88% | 40.71% | (0.063) |  |
| Married Cohabitant_  pre-migration | 69/132 | 53/113 | 0.008 | 0.012 |
|  | 52.27% | 53.10% | (0.064) |  |
| Divorced/widowed  pre -migration | 12/132 | 7/113 | -0.029 | -0.077 |
|  | 9.09% | 6.19% | (0.034) |  |
| Unemployed  pre-migration | 16/131 | 12/113 | -0.016 | -0.035 |
|  | 12.21% | 10.62% | (0.041) |  |
| Employed  pre-migration | 74/131 | 67/113 | 0.028 | 0.040 |
|  | 56.49% | 59.29% | (0.064) |  |
| Self-employed  pre-migration | 18/131 | 14/113 | -0.014 | -0.028 |
|  | 13.74% | 12.39% | (0.044) |  |
| Student pre-migration | 8/131 | 8/113 | 0.010 | 0.028 |
|  | 6.11 | 7.08% | (0.032) |  |
| Alone pre-migration | 11/131 | 11/112 | 0.014 | 0.035 |
|  | 8.40% | 9.82% | (0.037) |  |
| Spouse/Cohabitant  pre-migratio | 60/131 | 49/112 | -0.021 | -0.029 |
|  | 45.80% | 43.75% | 0.064 |  |
| Parents pre-migration | 50/131 | 45/112 | 0.020 | 0.029 |
|  | 38.17% | 40.18% | 0.063 |  |
| Others (including relatives) | 10/131 | 7/112 | -0.014 (0.033) | -0.038 |
| Age at departure in years: Mean (SD) | 27.836 (9.043) | 29.523 (10.185) | 1.687 (1.255) | **0.124** |
| Departure agreement | 95/124 | 83/111 | -0.018 | -0.030 |
|  | 76.61% | 74.77% | (0.056) |  |
| Balkan or Mediterranean route | 49/133 | 27/113 | -0.129 | **-0.200** |
|  | 36.84% | 23.89% | (0.059) |  |
| Eastern route | 24/133 | 32/113 | 0.103 | **0.173** |
|  | 18.05% | 28.32% | (0.053) |  |
| African route | 34/133 | 30/113 | 0.010 | 0.016 |
|  | 25.56% | 26.55% | (0.056) |  |
| Other route | 24/133 | 23/113 | 0.023 | 0.041 |
|  | 18.05% | 20.35% | (0.050) |  |
| Travel duration in month | 11.419 (20.587) | 9.223 (19.017) | -2.196 (2.784) | -0.078 |
| Present country is the final destination | 118/133 | 9/112 | 0.032 | 0.077 |
|  | (88.72%) | (91.96%) | (0.038) |  |
| Detention during travel | 36/101 | 32/109 | 0.019 | 0.029 |
|  | (27.48%) | (29.36%) | (0.059) |  |
| Detention duration in  months | 0.616 (1.827) | 0.828 (2.065) | 0.212 (0.248) | 0.077 |
| Travel alone | 18/133 | 25/113 | 0.086 | **0.159** |
|  | 13.53% | 22.12% | (0.048) |  |
| Travel with friends or  relatives | 70/133 | 52/113 | -0.066 | -0.093 |
|  | 52.63% | 46.02% | (0.064) |  |
| Migrants travel | 45/133 | 32/113 | -0.055 | -0.084 |
|  | 33.83% | 28.32% | (0.059) |  |
| ***Relatives in the country of origin*** | 113/131 | 100/112 | 0.030 | 0.065 |
|  | 86.26% | 89.29% | (0.042) |  |
| Sons/daughters | 14/133 | 14/113 | 0.019 | 0.041 |
|  | 10.53% | 12.39% | (0.041) |  |
| Partner: husband/wife | 12/133 | 12/113 | 0.016 | 0.038 |
|  | 9.02% | 10.62% | (0.038) |  |
| Parents | 63/133 | 56/113 | 0.022 | 0.031 |
|  | 47.37% | 49.56% | (0.064) |  |
| Other relatives | 82/133 | 75/113 | 0.047 | 0.069 |
|  | 61.65% | 66.37% | (0.062) |  |
| Friends | 25/133 | 25/113 | 0.033 | 0.058 |
|  | 18.80% | 22.12% | (0.052) |  |
| Which relative unreported | 9/133 | 9/113 | 0.012 | 0.032 |
|  | 6.77% | 7.96% | (0.033) |  |
| Asylum seeker | 71/133 | 62/113 | 0.015 | 0.021 |
|  | 53.38% | 54.87% | (0.064) |  |
| Humanitarian protection | 24/133 | 15/113 | -0.048 | -0.093 |
|  | 18.05% | 13.27% | (0.047) |  |
| Subsidiary protection | 6/133 | 7/113 | 0.017 | 0.053 |
|  | 4.51% | 6.19% | (0.029) |  |
| Political asylum | 24/133 | 22/113 | 0.014 | 0.026 |
|  | 18.05% | 19.47% | (0.050) |  |
| Other legal status | 7/133 | 7/113 | 0.009 | 0.028 |
|  | 5.26% | 6.19% | (0.030) |  |
| Length of stay in host  country in months:  Mean (SD) | 34.798 (24.034) | 34.308 (27.059) | -0.491(3.351) | -0.014 |
| Living alone  post-migration | 23/131 | 24/111 | 0.041 | 0.072 |
|  | 17.56% | 21.62% | (0.051) |  |
| Living with spouse post-migration | 48/131 | 44/111 | 0.030 | 0.043 |
|  | 36.64% | 39.64% | (0.063) |  |
| Living with parents post- migration | 7/131 | 3/111 | -0.026 | -0.095 |
|  | 5.34% | 2.70% | (0.026) |  |
| Living with others post-migration | 53/131 | 40/111 | -0.044 | -0.064 |
|  | 40.46% | 36.04% | (0.063) |  |
| Refugee center | 42/133 | 37/112 | 0.015 | 0.022 |
|  | 31.58 % | 33.04% | (0.060) |  |
| Rented apartment post-migration | 74/133 | 64/112 | 0.015 | 0.021 |
|  | 55.64% | 57.14% | (0.064) |  |
| Other accommodation  post-migration | 15/133 | 8/112 | -0.041 | **-0.101** |
|  | 11.28% | 7.14% | (0.037) |  |
| Accommodation found through government centers | 66/131 | 58/110 | 0.023 | 0.033 |
|  | 50.38% | 52.73 | (0.065) |  |
| Accommodation found through private  association | 35/131 | 29/110 | -0.004 | -0.006 |
|  | 26.72% | 26.36% | (0.057) |  |
| Accommodation found through facilitation of the hosting country | 17/131 | 15/110 | 0.007 | 0.014 |
|  | 12.98% | 13.64% | (0.044) |  |
| Accommodation found in other way | 13/131 | 8/110 | -0.027 | -0.067 |
|  | 9.92% | 7.27% | (0.037) |  |
| Unemployed  post-migration | 55/133 | 57/112 | 0.095 | **0.135** |
|  | 41.35% | 50.89% | (0.064) |  |
| Employed  post-migration | 28/133 | 14/112 | -0.086 | **-0.162** |
|  | 21.05% | 12.50% | (0.048) |  |
| Student  post-migration | 26/133 | 18/112 | -0.035 | -0.064 |
|  | 19.55% | 16.07% | (0.049) |  |
| Home maker  post-migration | 7/133 | 9/112 | 0.028 | 0.078 |
|  | 5.26% | 8.04% | (0.032) |  |
| Vocational training post-migration | 9/133 | 7/112 | -0.005 | -0.015 |
|  | 6.77% | 6.25% | (0.032) |  |
| Other occupation post-migration | 8/133 | 7/112 | 0.002 | 0.007 |
|  | 6.02% | 6.25% | (0.031) |  |
| ***Clinical variables*** |  |  |  |  |
| GHQ-12 | 5.414(2.422) | 5.301(2.104) | -0.113(0.292) | -0.035 |
| PCL-5 | 23.040(15.636) | 25.497(16.729) | 2.457(2.066) | **0.107** |
| PHQ-9 | 8.639(5.575) | 7.938(5.526) | -0.701(0.710) | -0.089 |
| WHO-5 | 46.466(24.315) | 45.881(23.242) | -0.585(3.049) | -0.017 |
| WHODAS | 0.154(0.140) | 0.153(0.145) | -0.001(0.018) | -0.005 |
| PSYCHLOPS | 13.930(4.241) | 14.339(3.734) | 0.409(0.528) | 0.072 |
| EQ-5D-3L | 0.690(0.285) | 0.702(0.297) | 0.012(0.037) | 0.028 |
| Observations | 133 | 113 | 246 | - |

**Table 2. Per-protocol analysis**

| **Variable** | **ETAU** | **SH+** | **Coef (CI)** | **p-value** |
| --- | --- | --- | --- | --- |
| **CONTINUOUS OUTCOMES** |  |  |  |  |
| **GHQ-12** |  |  |  |  |
| Screening (N=346) | 5.507(2.447) | 5.427(2.147) | - | - |
| 12 months (N=303): mixed | 2.930(3.298) | 1.718(2.502) | **-1.240 (-2.016; -0.465)** | **0.002** |
| 12 months LOCF(N=303) | 3.073(3.312) | 2.028(2.827) | **-0.983 (-1.663; -0.303)** | **0.005** |
| **PCL-5** |  |  |  |  |
| Baseline (N=346) | 22.765(16.239) | 24.744(16.242) | - |  |
| 12 months (N=302): mixed | 14.606(15.306) | 12.937(15.569) | -3.307 (-7.123; 0.568) | 0.094 |
| 12 months LOCF(N=302) | 16.350(15.023) | 14.300(15.210) | -2.579 (-5.751; 0.592) | 0.111 |
| **PHQ-9** |  |  |  |  |
| Baseline (N=346) | 8.384(5.546) | 8.077(5.8887) | - | - |
| 12 months (N=301): mixed | 5.810(6.045) | 3.776(4.221) | **-1.839 (-3.182; -0.497)** | **0.007** |
| 12 months LOCF(N=301) | 6.335(5.917) | 4.563(5.170) | **-1.522 (-2.735; -0.309)** | **0.014** |
| **WHO-5** |  |  |  |  |
| Baseline(N=346) | 47.354(24.984) | 45.750(23.478) | - | - |
| 12 months (N=302): mixed | 52.157(27.248) | 62.087(24.597) | **10.761 (4.047; 17.476)** | **0.002** |
| 12 months LOCF(N=302) | 50.333(26.673) | 60.436(23.904) | **10.375 (4.511; 16.239)** | **0.001** |
| **WHODAS** |  |  |  |  |
| Baseline(N=346) | 0.152(0.144) | 0.157(0.131) | - | - |
| 12 months (N=302): mixed | 0.088(0.107) | 0.075(0.110) | -0.018 (-0.045; 0.010) | 0.202 |
| 12 months LOCF(N=302) | 0.092(0.123) | 0.076(0.104) | -0.015 (-0.037; 0.008) | 0.206 |
| **PSYCHLOPS** |  |  |  |  |
| Baseline(N=323) | 13.925(4.396) | 14.200(3.468) | - | - |
| 12 months (N=292): mixed | 10.758(5.431) | 10.123(5.249) | -0.561 (-2.079; 0.956) | 0.468 |
| 12 months LOCF(N=292) | 10.959(5.432) | 10.216(5.319) | -0.721 (-1.968; 0.526) | 0.256 |
| **PMLD** |  |  |  |  |
| Post-intervention* (*n*=283) | 25.587(12.846) | 24.174(12.199) | - | - |
| 12 months: mixed (N=262) | 17.811(11.833) | 17.692(12.613) | -0.797 (-3.643; 2.049) | 0.583 |
| 12 months LOCF (N=262) | 18.844(11.489) | 18.208(11.736) | -0.692 (-3.156; 1.772) | 0.581 |
| **EQ-5D-3L** |  |  |  |  |
| Baseline (N=345) | 0.706(0.276) | 0.696(0.281) | - | - |
| 12 months: mixed (N=262) | 1.460(1.542) | 1.464(1.587) | 0.003 (-0.472; 0.479) | 0.990 |
| 12 months LOCF (N=262) | 1.277(1.371) | 1.288(1.411) | 0.0001 (-0.332; 0.332) | 0.999 |
| **Legend:** Values in bold highlight statistically significant differences. n indicates the number of observations at the first available measurement, while N represents the number of individuals used in regression.  *Not measured at baseline. | | | | |

**Table 3. Analysis with no imputation**

| **CONTINUOUS OUTCOMES** | **Coef** | **CI** | **p-value** |
| --- | --- | --- | --- |
| GHQ-12 | **-1.020** | **(-1.720; -0.320)** | **0.004** |
| PCL-5 | -3.144 | (-6.512; 0.223) | 0.067 |
| PHQ-9 | **-1.567** | **(-2.807; -0.328)** | **0.013** |
| WHO-5 | **9.777** | **(3.784; 15.770)** | **0.001** |
| WHODAS | -0.018 | (-0.041; 0.005) | 0.132 |
| PSYCHLOPS | -0.686 | (-2.087; 0.715) | 0.337 |
| PMLD | -0.012 | (-2.645; 2.622) | 0.993 |

**Table 4. Results controlling for imbalances at baseline**

| **BINARY OUTCOMES** | **RR** | **CI** | **p-value** |
| --- | --- | --- | --- |
| MINI | 0.751 | (0.457; 1.235) | 0.260 |
| **CONTINUOUS OUTCOMES** | **Coef** | **CI** | **p-value** |
| GHQ-12 | -0.636 | (-1.319; 0.047) | 0.068 |
| PCL-5 | -1.980 | (-4.912; 0.953) | 0.185 |
| PHQ-9 | **-1.248** | **(-2.469; -0.026)** | **0.045** |
| WHO-5 | **9.686** | **(4.446; 14.926)** | **<0.001** |
| WHODAS | -0.012 | (-0.033; 0.009) | 0.266 |
| PSYCHLOPS | -0.414 | (-1.595; 0.767) | 0.491 |
| PMLD | 0.168 | (-2.234; 2.570) | 0.891 |
| EQ-5D-3L | -0.225 | (-0.549; 0.099) | 0.173 |

**Table 5. Balance of clinical and sociodemographic variables between Lost-to-follow-up (LTFU) and non-LTFU (completers)**

| **Clinical Variable** | **Mean completers** | **Mean LTFU** | **Difference (SE)** | **Standardized Mean Difference** |
| --- | --- | --- | --- | --- |
| GHQ-12 | 5.362 | 5.796 | 0.434 | **0.133** |
|  | (2.277) | (2.347) | (0.216) |  |
| PCL-5 | 24.168 | 23.226 | -0.943 | -0.041 |
|  | (16.161) | (16.497) | (1.527) |  |
| PHQ-9 | 8.317 | 8.563 | 0.246 | 0.031 |
|  | (5.552) | (5.827) | (0.532) |  |
| WHO-5 | 46.197 | 48.008 | 1.811 | 0.053 |
|  | (23.782) | (24.881) | (2.274) |  |
| WHODAS | 0.153 | 0.147 | -0.007 | -0.034 |
|  | (0.142) | (0.143) | (0.013) |  |
| PSYCHLOPS | 14.118 | 13.888 | -0.230 | -0.039 |
|  | (4.013) | (4.421) | (0.406) |  |
| EQ-5D-3L | 0.696 | 0.728 | 0.032 | 0.083 |
|  | (0.290) | (0.262) | (0.026) |  |
| **Sociodemographic variables** |  |  |  |  |
| Mean age (SD), years | 32.654 | 31.784 | -0.870 | -0.060 |
|  | (9.849) | (10.547) | (0.953) |  |
| Gender | 68/246. | 66/213 | 0.033 | 0.052 |
|  | 27.64% | 30.99% | (0.043) |  |
| *Country of origin* |  |  |  |  |
| Afghanistan | 42/246 | 24/213 | -0.058 | **-0.118** |
|  | (17.07%) | (11.27%) | (0.033) |  |
| Iraq | 46/246 | 37/213 | -0.013 | -0.024 |
|  | (18.70%) | (17.37%) | (0.036) |  |
| Nigeria | 59/246 | 55/213 | 0.018 | 0.030 |
|  | (23.98%) | (25.82%) | (0.041) |  |
| Pakistan | 16/246 | 25/213 | 0.052 | **0.129** |
|  | (6.50%) | (11.74%) | (0.027) |  |
| Syria | 72/246 | 58/213 | -0.020 | -0.032 |
|  | (29.27% ) | (27.23%) | (0.042) |  |
| Other_country | 11/246 | 14/213 ( | 0.021 | 0.065 |
|  | (4.47%) | (6.57%) | (0.021) |  |
| Mean relatives (SD), *n* | 1.728 | 1.305 | -0.422 | **-0.137** |
|  | (2.331) | (2.036) | (0.206) |  |
| Mean children (SD), *n* | 1.523 | 1.170 | -0.353 | **-0.118** |
|  | (2.331) | (1.860) | (0.200) |  |
| Mean education (SD), years | 10.192 | 10.433 | 0.241 | 0.033 |
|  | (5.411) | (4.899) | (0.498) |  |
| *Type of education* |  |  |  |  |
| Illiterate | 23/242 | 16/211 | -0.019 | -0.049 |
|  | (9.50%) | (7.58%) | (0.026) |  |
| Primary | 98/242 | 96/211 | 0.050 | 0.071 |
|  | (40.50%) | (45.50%) | (0.047) |  |
| High_School | 64/242 | 62/211 | 0.029 | 0.046 |
|  | (26.45%) | (29.38%) | (0.042) |  |
| University | 55/242 | 37/211 | -0.052 | -0.092 |
|  | (22.73%) | (17.54%) | (0.038) |  |
| *Travel route* |  |  |  |  |
| Balkan | 76/246 | 68/213 | 0.010 | 0.016 |
|  | (30.89%) | (31.92%) | (0.044) |  |
| Eastern | 56/246 | 40/213 | -0.040 | -0.069 |
|  | (22.76%) | (18.78%) | (0.038) |  |
| African | 64/246 | 61/213 | 0.026 | 0.042 |
|  | (26.02%) | (28.64%) | (0.042) |  |
| Other_route | 47/246 | 42/213 | 0.006 | 0.011 |
|  | (19.11%) | (19.72%) | (0.037) |  |
| Travel duration in month Mean (SD) | 10.401 | 10.535 | 0.134 | 0.005 |
|  | (19.857) | (21.681) | (2.098) |  |
| Observations | 246 | 213 | 459 |  |

**Table 6. Frequency of health service use during the study**

|  | ETAU  (N=135) | SH+  (N=114) | Total (N=249) | Chi-square/Fisher’s exact test* |
| --- | --- | --- | --- | --- |
|  | Use n (%) | Use n (%) | Use n (%) | P |
| Primary health care | 50 (37.04) | 42 (36.84) | 92 (36.95) | 0.975 |
| Community mental health care | 2 (1.48) | 2 (1.75) | 4 (1.61) | 1.000 |
| Any outpatient treatment | 20 (14.81) | 12 (10.53) | 32 (12.85) | 0.314 |
| General hospital inpatient treatment | 3 (2.22) | 6 (5.26) | 9 (3.61) | 0.348 |
| Any medications | 22 (16.30) | 15 (13.16) | 37 (14.86) | 0.488 |
| Total | 72 (53.33) | 54 (47.37) | 126 (50.60) | 0.348 |
| **Legend:** *Fisher’s exact test was adopted if at least one cell had an expected frequency below 5; Chi-square test was performed otherwise. | | | | |

**Test of interactions**

In separate regression models for each possible moderator (i.e., the variables related to gender, education in years, age, length of stay in the host country, country of origin, and recruiting center) considering, as outcome variable, the binary outcomes and, as regressors, intervention allocation, the potential moderator and their interaction, none of the interactions reached the conventional statistical threshold at 12 month follow-up. By performing seemingly unrelated regression on continuous outcomes, a global test on all interactions of intervention status turned out to be statistically significant (p-value 0.004). Single regressions were tested and found global statistical significance of all interactions only in the regression having PSYCHLOPS as outcome (p-value 0.042). This analysis found that study center (p-value 0.029) and gender (p-value 0.008) emerged as statistically significant moderators. In particular, a statistically significant protective effect of Self-Help Plus on PSYCLOPS score at 12-months emerged for people recruited in York and Ulm study sites and for women.

**Figure 1: Estimated effect of SH+ on PSYCHLOPS at 12 months per recruitment site**


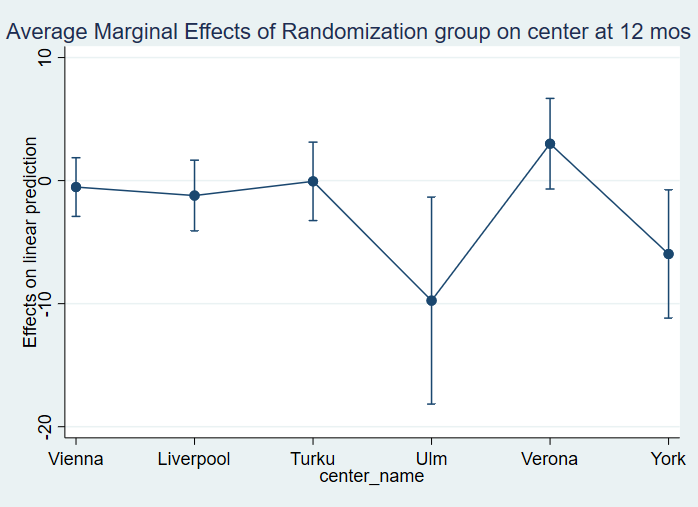


**Figure 2: Estimated effect of SH+ on PSYCHLOPS at 12 months per gender**


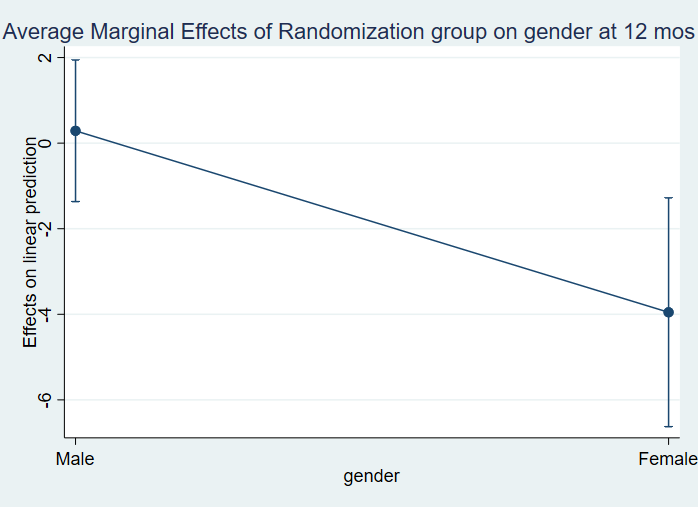


**List of adverse events**

| Event | Allocation arm |
| --- | --- |
| 1. Increase in emotional distress because the participant is no longer in reception center. | ETAU |
| 2. Increase in suicidal thoughts - major depressive episode not related to research – referred to healthcare services | SH+ |
| 3. Increase in emotional distress: the subject reports sensation of heat all over the body as if it was on fire | ETAU |


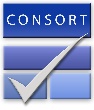
CONSORT 2010 checklist of information to include when reporting a randomised trial

| Section/Topic | Item No | Checklist item | Reported on page No |
| --- | --- | --- | --- |
| Title and abstract | | | |
|  | 1a | Identification as a randomised trial in the title | 1 |
|  | 1b | Structured summary of trial design, methods, results, and conclusions (for specific guidance see CONSORT for abstracts) | 3 |
| Introduction | | | |
| Background and objectives | 2a | Scientific background and explanation of rationale | 5-6 |
|  | 2b | Specific objectives or hypotheses | 6 |
| Methods | | | |
| Trial design | 3a | Description of trial design (such as parallel, factorial) including allocation ratio | 6-7 |
|  | 3b | Important changes to methods after trial commencement (such as eligibility criteria), with reasons | N/A |
| Participants | 4a | Eligibility criteria for participants | 7-8 |
|  | 4b | Settings and locations where the data were collected | 6-7 |
| Interventions | 5 | The interventions for each group with sufficient details to allow replication, including how and when they were actually administered | 8-9-10 |
| Outcomes | 6a | Completely defined pre-specified primary and secondary outcome measures, including how and when they were assessed | 10-11-12 |
|  | 6b | Any changes to trial outcomes after the trial commenced, with reasons | N/A |
| Sample size | 7a | How sample size was determined | 12 |
|  | 7b | When applicable, explanation of any interim analyses and stopping guidelines | N/A |
| Randomisation: |  |  |  |
| Sequence generation | 8a | Method used to generate the random allocation sequence | 10 |
|  | 8b | Type of randomisation; details of any restriction (such as blocking and block size) | 10 |
| Allocation concealment mechanism | 9 | Mechanism used to implement the random allocation sequence (such as sequentially numbered containers), describing any steps taken to conceal the sequence until interventions were assigned | 10 |
| Implementation | 10 | Who generated the random allocation sequence, who enrolled participants, and who assigned participants to interventions | 10 |
| Blinding | 11a | If done, who was blinded after assignment to interventions (for example, participants, care providers, those assessing outcomes) and how | 10 |
|  | 11b | If relevant, description of the similarity of interventions | N/A |
| Statistical methods | 12a | Statistical methods used to compare groups for primary and secondary outcomes | 12-15 |
|  | 12b | Methods for additional analyses, such as subgroup analyses and adjusted analyses | 12-15 |
| Results | | | |
| Participant flow (a diagram is strongly recommended) | 13a | For each group, the numbers of participants who were randomly assigned, received intended treatment, and were analysed for the primary outcome | 15-16 |
|  | 13b | For each group, losses and exclusions after randomisation, together with reasons | 15 |
| Recruitment | 14a | Dates defining the periods of recruitment and follow-up | 15-16 |
|  | 14b | Why the trial ended or was stopped | N/A |
| Baseline data | 15 | A table showing baseline demographic and clinical characteristics for each group | 16-17-18 |
| Numbers analysed | 16 | For each group, number of participants (denominator) included in each analysis and whether the analysis was by original assigned groups | 16-17-18 |
| Outcomes and estimation | 17a | For each primary and secondary outcome, results for each group, and the estimated effect size and its precision (such as 95% confidence interval) | 16-17-18 |
|  | 17b | For binary outcomes, presentation of both absolute and relative effect sizes is recommended | 16-17-18 |
| Ancillary analyses | 18 | Results of any other analyses performed, including subgroup analyses and adjusted analyses, distinguishing pre-specified from exploratory | 17-18 |
| Harms | 19 | All important harms or unintended effects in each group (for specific guidance see CONSORT for harms) | 16-17 |
| Discussion | | | |
| Limitations | 20 | Trial limitations, addressing sources of potential bias, imprecision, and, if relevant, multiplicity of analyses | 20-21-22 |
| Generalisability | 21 | Generalisability (external validity, applicability) of the trial findings | 22-23 |
| Interpretation | 22 | Interpretation consistent with results, balancing benefits and harms, and considering other relevant evidence | 19-20 |
| Other information | | |  |
| Registration | 23 | Registration number and name of trial registry | 7 |
| Protocol | 24 | Where the full trial protocol can be accessed, if available | 7 |
| Funding | 25 | Sources of funding and other support (such as supply of drugs), role of funders | 23 |
